# Dose of colistin: a work in progress?

**DOI:** 10.1186/s13054-015-0743-x

**Published:** 2015-02-16

**Authors:** Monica Rocco, Luca Montini, Gennaro De Pascale, Massimo Antonelli

**Affiliations:** Department of Medicine-Surgical Science and Translational medicine, Sapienza University, Via di Grottarossa 1035, Rome, 00135 Italy

**Keywords:** ■■■

We thank Rashid and colleagues [1] and Honoré and colleagues [2] for their comments regarding our article on risk factors for acute kidney injury in patients receiving colistin or other nephrotoxic antimicrobials [3].

It is correct that we did not specifically report urine output in the text, but it was obviously included in the RIFLE (Risk, Injury, Failure, Loss of kidney function, and End-stage kidney disease) criteria reported in Table two [3].

We agree that the colistin methanesulfonate pharmacokinetics have been better studied recently, and it has become clear that high doses are required for treating multidrug-resistant Gram-negative bacilli infection, including the loading dose (which has changed from 4 to 9 million IU) and the dose interval (which has changed from three to two times a day) [4]. We agree with the authors’ concerns about the adequacy of colistin dosages adopted in our cohort (130,000 IU/kg of ideal body weight, modified according to renal function), but these doses were commonly adopted, especially in patients with renal impairment [5]. Indeed, the development of new high-performance liquid chromatography assays that allow clinicians to measure the concentrations of colistimethate and colistin separately has shown that colistin clearance is due mainly to non-renal mechanisms that are still unclear. It is of great interest to note, as reported recently by Honoré and colleagues [6], that patients with multidrug-resistant infections can receive even higher doses of colistin during continuous renal replacement therapy, as colistin methanesulfonate is continuously filtered and absorbed by dialysis membrane [7]. Hence, even higher doses may be needed in patients receiving continuous renal replacement therapy than in patients with normal renal function. The growing evidence in favor of a higher dosage of colistin requires further clinical studies.
